# Different accelerated corneal collagen cross-linking treatment modalities in progressive keratoconus

**DOI:** 10.1186/s40662-019-0141-6

**Published:** 2019-06-03

**Authors:** Ahmet Kirgiz, Mustafa Eliacik, Yusuf Yildirim

**Affiliations:** 1 Department of Ophthalmology, University of Health Sciences, Bagcilar Training and Research Hospital, Istanbul, Turkey; 2 Department of Ophthalmology, Istanbul Medipol University, Istanbul, Turkey; 3 University of Health Sciences, Beyoglu Eye Training and Research Hospital, Istanbul, Turkey

**Keywords:** Accelerated crosslinking, Corneal collagen CXL, High order aberrations, Keratoconus, Topography

## Abstract

**Background:**

To compare the outcomes of two different protocols of accelerated corneal crosslinking (CXL) on visual, corneal high order aberrations (HOA) and topographic parameters in patients with progressive keratoconus.

**Methods:**

In this prospective comparative study, sixty-six eyes of 66 patients with progressive keratoconus were divided into two groups; 37 eyes in Group 1 received 18 mW/cm^2^ for five minutes, and 29 eyes in Group 2 were treated with 9 mW/cm^2^ for 10 min. The uncorrected distant visual acuity (UCVA), best-corrected distant visual acuity (BCVA), corneal HOAs and topography parameters were measured preoperatively and postoperatively at the end of 12 months. The data for the two groups were compared statistically.

**Results:**

The mean UCVA and BCVA were significantly improved at the postoperative 12 months compared with the preoperative values in both groups (P < 0.05 for all). A significant improvement in corneal HOAs was observed in both groups (P < 0.05 for all). The change in corneal coma value was significantly higher in Group 2 (P < 0.05). The change in keratometric values K1, K2, AvgK and maximum keratometry (AKf) were significantly higher in Group 2 (P < 0.05 for all). The regression model showed that the most important factor predicting the change in AKf was the type of CXL (β = − 0.482, *P* = 0.005).

**Conclusions:**

Accelerated CXL using 10 min of UVA irradiance at 9 mW/cm^2^ showed better topographic improvements and coma values than five minutes of UVA irradiance at 18 mW/cm^2^ independent of keratoconus severity.

## Background

Keratoconus is a bilateral ectatic disease characterized by progressive thinning of the cornea, paracentral steepening and irregular astigmatism with onset at puberty in most cases. Although spectacle correction and rigid gas-permeable contact lenses are the main treatment for mild cases, surgery becomes necessary because of the progressive nature of the disease.

In 2003, an encouraging study demonstrating a successful parasurgical method for halting keratoconus progression was published by Wollensak et al. [1]. The basic principle of this method is the chemical interaction of ultraviolet (UV) A and riboflavin to induce covalent bond formation between collagen fibers of the cornea. In this way, the stiffness and rigidity of the cornea are provided. In this study, a conventional crosslinking (CXL) method was described as a 3 mW/cm^2^ irradiance with 370 nm UVA light with a total dose of 5.4 J/cm^2^ applied to the cornea after a 30 min installation of riboflavin.

The conventional method of CXL has become an important treatment of keratoconus since it was introduced [2]. However, the length of the standard treatment time has led to the development of accelerated crosslinking methods [3]. The effectiveness of accelerated CXL protocols began to be compared with conventional methods over time [4–8]. Despite these comparative studies with standard protocol, the studies comparing different accelerated CXL protocols with each other are limited [9–11].

In this study, we compared the visual, corneal high order aberrations (HOA) and topographic parameters of accelerated CXL protocols using five minutes of UVA irradiance at 18 mW/cm^2^ and 10 min of UVA irradiance at 9 mW/cm^2^. This is the first study that verifies if preoperative data could be a predictive factor of success of two different accelerated CXL methods on progressive keratoconus.

## Methods

In this prospective comparative study, patients with progressive keratoconus who underwent two different irradiance doses of accelerated CXL in Bagcilar Education and Research Hospital, Istanbul, Turkey, were included. Between January 2016 and March 2017, 80 consecutive patients who were diagnosed with progressive keratoconus were randomly given accelerated CXL with an irradiance of 18 mW/cm^2^ for five minutes (Group 1) or an irradiance of 9 mW/cm^2^ for 10 min (Group 2). The patients were equally placed into the groups for randomization with the help of a computer-generated random number table and those who were followed regularly for one year were included in this study (37 eyes for Group 1 and 29 eyes for Group 2). The study was carried out in accordance with the principles stated in the Helsinki Declaration and approved by the Clinical Research Ethics Committee of Bagcilar Education and Research Hospital (Project # 2015/233). Written informed consent was obtained from all patients before participation in the study.

The keratoconus progression was evaluated as follows: an increase of 1.00 diopter (D) or more in the steepest keratometry (K) measurement, manifest cylinder or manifest refraction spherical equivalent or the loss of at least two lines of best-corrected distance visual acuity (BCVA) in the past 12 months. Patients were excluded if they were older than 40 years of age and had a corneal thickness less than 400 μm at the thinnest point, apical corneal scarring, hydrops or severe dry eye, a history of corneal surgery, pregnant or lactating women throughout the study. Wearers of hard contact lenses discontinued their use three weeks before any assessment and surgery.

All the patients had a complete ophthalmic examination including the uncorrected distant visual acuity (UCVA) and BCVA using a Snellen chart, manifest refraction, slit-lamp biomicroscopy and dilated fundoscopy. Topographical measurements and corneal HOAs were obtained using a placido disk topography with Sheimpflug tomography of the anterior segment (Sirius, Costruzione Strumenti Oftalmici, Italy) according to the manufacturer’s guideline by the same trained examiner. Corneal topographic parameters as well as the corneal total HOAs, spherical aberrations, coma and trefoil values (anterior cornea at 6 mm zone) were recorded from the topography. All patients underwent these investigations in the preoperative period and at one, three, six, and twelve months postoperatively. Visual acuity based on the Snellen chart was converted to LogMAR units for statistical analysis. Patients were divided into two subgroups: those with a maximum keratometry (Kmax) value of < 58.0 D defined as mild to moderate keratoconus and Kmax of ≥ 58.0 D as advanced keratoconus according to the baseline Kmax in both groups [12].

The demarcation line depth was measured by using anterior segment optical coherence tomography (AS-OCT) (Visante, Carl Zeiss, Dublin, CA) at 1 month postoperatively to allow for an accurate comparison. The measurements were taken from the center of the cornea where the reflection of the demarcation line was visible. It was manually marked with the help of a caliper tool provided by the software of the device.

### Surgical technique

Accelerated CXL has been performed on all patients. Initially, a topical anesthetic agent, proparacaine 0.5%, (Alcaine; Alcon Laboratories, Inc.) was administered and 8 mm central corneal epithelium was removed with a blunt spatula. Riboflavin with dextran (0.1% riboflavin in 20% dextran, Medicross, Germany) solution was then administered topically every two minutes for 20 min in both groups. After the installation of the riboflavin as a photosensitizer, the cornea was exposed to a UV-A 365 nm light for five minutes at an irradiance of 18 mW/cm^2^ in Group 1 and 10 min at an irradiance of 9 mW/cm^2^ in Group 2 (Peschke Meditrade, GmbH, Switzerland). Riboflavin was instilled every minute during the UV-A exposure in Group 1 and every two minutes in Group 2. After treatment, the eye surface was washed with 20 mL of a balanced salt solution, medicated with antibiotic eye drops (moxifloxacin 0.5%, Vigamoxª; Alcon Co., Inc.) and dressed with a bandage soft contact lens until the closure of epithelial defect. Topical moxifloxacin eye drops (four times per day for one week; Vigamox; Alcon Co., Inc.), artificial tears (four times per day for one month) and loteprednol etabonate ophthalmic suspension 0.5% (four times per day two weeks after epithelial healing; Lotemax; Bausch & Lomb) were administered postoperatively.

### Statistical analysis

Statistical package for the social sciences, version 22 (SPSS, Chicago, IL, USA), was used to analyze the data. The Kolmogorov-Smirnov test was performed to test distribution of the sample means. The categorical variables were compared between the groups using the χ^2^ test. The Student’s *t*-test was applied between the groups and paired *t*-test was used for independent variables. Spearman’s correlational and regression analysis was applied to predict change in Kmax as independent variables (age, sex, treatment type and preoperative measures). A *P* value less than 0.05 was considered significant.

## Results

In total, 66 eyes of 66 patients with a mean age of 24.5 ± 6.36 years (range: 14 to 38 years) were evaluated in this study. Thirty-seven eyes (Group 1) of 37 patients (21 males, 16 females) were treated with 18 mW/cm^2^ accelerated CXL for five minutes and 29 eyes (Group 2) of 29 patients (13 males, 16 females) were treated with 9 mW/cm^2^ accelerated CXL for 10 min. The mean ages of Groups 1 and 2 were 23.59 ± 6.85 and 25.66 ± 5.57 years, respectively. There were no significant differences between the groups with respect to age and sex (*P* = 0.194 and *P* = 0.336, respectively).

The preoperative values and postoperative alterations after 12 months in visual acuities, corneal HOAs and topographic findings are summarized in Tables 1, 2, and 3. There was no significant difference between the groups in terms of all preoperative values (*P* > 0.05). The UCVA and BCVA were significantly improved at 12 months postoperative compared with preoperative values in both groups (*P* < 0.05 for all). Corneal total HOAs, spherical aberrations, coma and trefoil values were significantly improved at the postoperative 12 months compared with the preoperative values in both groups (*P* < 0.05 for all). K1, K2 and AvgK values did not differ significantly in Group 1 (*P* > 0.05), but the difference in K2 and AvgK was significant in Group 2 (*P* = 0.0001). There was a significant decrease in the maximum keratometry (AKf or Kmax) in both groups (*P* = 0.005 and *P* = 0.001, respectively). The thinnest point of the cornea (Thin), corneal volume and the central corneal thickness (CCT) decreased significantly at 12 months in both groups (*P* < 0.05 for all).

**Table 1:** Comparison of visual acuities and corneal topographic findings between the two groups

		Group 1 (*n* = 37)	Group 2 (*n* = 29)	P
UCVA (LogMAR)	Preop	0.581 ± 0.341	0.662 ± 0.277	0.213
	12-month postop	0.449 ± 0.293	0.572 ± 0.299	0.059
	Mean change	0.13 ± 0.22	0.09 ± 0.21	0.510
	P*	**0.001**	**0.031**	
BCVA (LogMAR)	Preop	0.378 ± 0.237	0.421 ± 0.206	0.281
	12-month postop	0.281 ± 0.185	0.283 ± 0.161	0.936
	Mean change	0.10 ± 0.13	0.14 ± 0.15	0.137
	P*	**0.0001**	**0.0001**	
Cyl (D)	Preop	3.46 ± 1.49	3.51 ± 1.54	0.899
	12-month postop	3.48 ± 1.67	3.51 ± 1.75	0.937
	Mean change	−0.02 ± 0.57	0.00 ± 0.62	0.902
	P*	0.862	0.986	
Thin (μm)	Preop	463.51 ± 38.74	458.72 ± 37.11	0.613
	12-month postop	443.51 ± 46.79	418.14 ± 43.35	**0.027**
	Mean change	20.00 ± 20.76	40.59 ± 25.45	**0.0001**
	P*	**0.0001**	**0.0001**	
Vol (mm^3^)	Preop	56.62 ± 3.56	55.87 ± 4.15	0.434
	12-month postop	55.58 ± 3.93	54.59 ± 4.29	0.331
	Mean change	1.03 ± 1.62	1.28 ± ± .75	0.561
	P*	**0.0001**	**0.018**	
CCT (μm)	Preop	482.16 ± 39.52	474.1 ± 38.82	0.410
	12-month postop	471.05 ± 46.19	451.59 ± 41.84	0.081
	Mean change	11.11 ± 18.64	22.52 ± 21.42	**0.008**
	P*	**0.001**	**0.0001**	

*UCVA* = uncorrected visual acuity; *BCVA* = best-corrected visual acuity; *Cyl* = topographic cylindrical value; *Thin* = thinnest point of cornea; *Vol* = corneal volume; *CCT* = central corneal thickness; *D* = diopters

P: Paired t-test; P*: Student’s t-test; Values in bold are significant (*P* < 0.05)

**Table 2:** Comparison of keratometry readings and symmetry indices between the two groups

		Group 1 (*n* = 37)	Group 2 (*n* = 29)	*P*
K1 (D)	Preop	45.64 ± 2.8	45.88 ± 2.3	0.708
	12-month postop	45.71 ± 2.91	45.30 ± 3.02	0.580
	Mean change	−0.07 ± 0.72	0.58 ± 2.03	**0.001**
	P*	0.559	0.135	
K2 (D)	Preop	49.10 ± 3.56	49.39 ± 3.06	0.727
	12-month postop	49.19 ± 3.63	48.47 ± 3.27	0.408
	Mean change	−0.09 ± 0.79	0.92 ± 1.22	**0.0001**
	P*	0.506	**0.0001**	
AvgK (D)	Preop	47.3 ± 3.09	47.56 ± 2.56	0.715
	12-month postop	47.37 ± 3.15	46.63 ± 2.76	0.322
	Mean change	−0.08 ± 0.69	0.93 ± 1.14	**0.0001**
	P*	0.513	**0.0001**	
AKf (D)	Preop	56.38 ± 5.26	56.29 ± 5.34	0.949
	12-month postop	55.59 ± 4.98	54.44 ± 5.12	0.364
	Mean change	0.79 ± 1.59	1.85 ± 1.58	**0.003**
	P*	**0.005**	**0.001**	
AKb (D)	Preop	80.9 ± 10.76	80.95 ± 12.09	0.985
	12-month postop	84.61 ± 11.94	85.9 ± 12.64	0.672
	Mean change	−3.71 ± 6.06	−4.95 ± 4.35	0.108
	P*	**0.001**	**0.0001**	
SIf (D)	Preop	6.48 ± 3.74	6.6 ± 3.63	0.900
	12-month postop	6.14 ± 3.57	5.72 ± 3.44	**0.631**
	Mean Change	0.34 ± 0.93	0.88 ± 0.83	**0.008**
	P*	**0.031**	**0.0001**	
SIb (D)	preop	1.67 ± 0.84	1.63 ± 0.82	0.845
	12-month postop	1.75 ± 0.79	1.7 ± 0.85	0.796
	Mean change	−0.08 ± 0.26	−0.06 ± 0.29	0.999
	P*	0.091	0.254	

*K1* = flat keratometry; *K2* = steep keratometry; *AvgK* = average keratometry; *AKf* = apical keratoscopy front (Kmax); *AKb* = apical keratoscopy back; *SIf* = symmetry index front; *SIb* = symmetry index back; *D* = Diopters

*P*: Paired t test; P*: Student’s t-test; Values in bold are significant (*P* < 0.05)

**Table 3:** Preoperative and 12-month postoperative corneal high order aberrations

HOAs 6-mm	Group 1 *n* = 37	Group 2 *n* = 29	*P*
Total (RMS, μm)			
Preoperative	4.60 ± 2.0	4.66 ± 2.06	0.82
12 months postoperative	4.12 ± 1.78	4.08 ± 2.01	0.68
Mean difference	0.48 ± 0.26	0.58 ± 0.30	0.08
P*	**0.04**	**0.04**	
Spherical (RMS, μm)			
Preoperative	0.56 ± 0.38	0.58 ± 0.46	0.72
12 months postoperative	0.48 ± 0.41	0.49 ± 0.37	0.80
Mean difference	0.08 ± 0.06	0.08 ± 0.05	0.81
P*	**0.02**	**0.01**	
Coma (RMS, μm)			
Preoperative	4.28 ± 2.06	4.44 ± 1.92	0.16
12 months postoperative	3.82 ± 1.95	3.76 ± 1.81	0.32
Mean difference	0.46 ± 0.18	0.68 ± 0.21	**0.04**
P*	**0.02**	**< 0.01**	
Trefoil (RMS, μm)			
Preoperative	0.56 ± 0.36	0.59 ± 0.42	0.60
12 months postoperative	0.42 ± 0.38	0.44 ± 0.40	0.42
Mean difference	0.14 ± 0.09	0.15 ± 0.10	0.22
P*	**0.03**	**0.02**	

*HOAs* = higher order aberrations; *RMS* = root mean square

*P:* Paired t test; P*: Student’s t-test; Values in bold are significant (*P* < 0.05)

The mean changes for each parameter from baseline and postoperative 12 months were compared between the two groups (Tables 1, 2, and 3). The changes in UCVA and BCVA did not differ significantly in a statistical way between the two groups (*P* = 0.510 and *P* = 0.137, respectively). The changes in corneal total HOA, spherical aberrations and trefoil values did not differ significantly in a statistical way between the two groups (*P* = 0.08, *P* = 0.81 and *P* = 0.22, respectively). Statistically, the change in corneal coma value was significantly higher in Group 2 (*P* < 0.05). The change in keratometric values K1, K2, AvgK and AKf were significantly higher in Group 2 (*P* < 0.05 for all). The change in symmetry index back (SIb) did not differ between the two groups (*P* = 0.99), but the change in symmetry index front (SIf) was significantly higher in Group 2 (*P* = 0.008). The change in Thin and CCT was also higher in Group 2 (*P* = 0.0001 and *P* = 0.008, respectively).

**Fig. 1:**
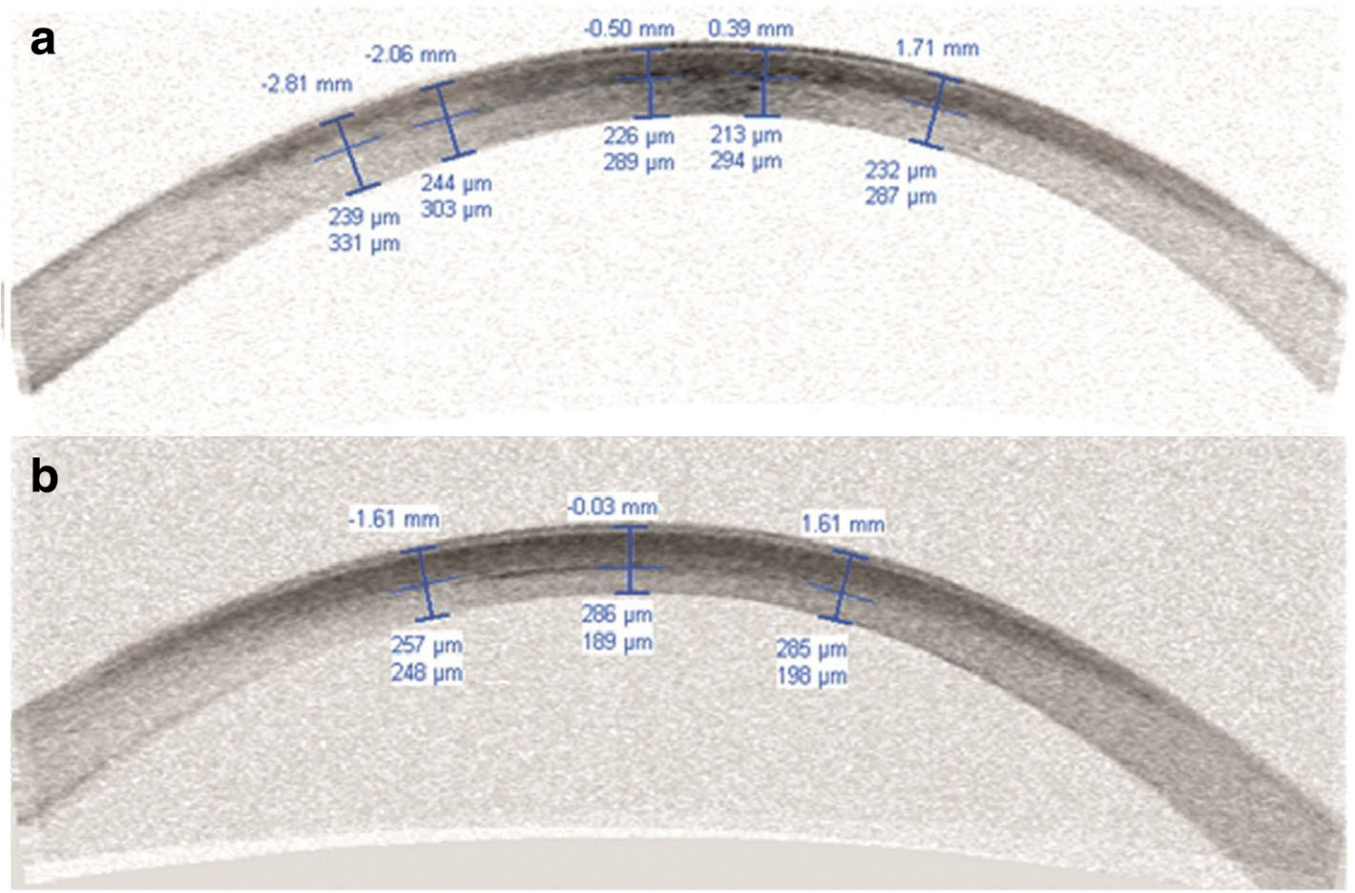
AS-OCT shows the demarcation lines of two patients treated with accelerated CXL ((**a**) 5 min 18 mW/cm^2^; 213 μm depth, (**b**) 10 min 9 mW/cm^2^; 286 μm depth). It is clearly observed that the demarcation line is deeper in 10 min protocol

The mean depth of demarcation line was 228.03 ± 36.74 μm (min 169 μm to max 305 μm) in Group 1 and 279.86 ± 31.95 μm (min 208 μm to max 355 μm) in Group 2. The difference between the two groups was significant (*P* = 0.001). The fig. 1 shows examples of demarcation lines from both groups.

Subgroup analysis showed no difference in terms of the mean change in UCVA and BCVA between the mild to moderate and advanced keratoconus in both groups (*P* > 0.05 for all). However, both subgroups showed a significant decrease in the mean changes in K2 and AvgK in Group 2, with K1 in advanced cases (*P* < 0.05 for all). AKf also showed an evident but not significant decrease in both subgroups in Group 2 (*P* > 0.05) (Table 4).

**Table 4:** Changes in parameters from baseline after accelerated crosslinking with 18 mW/cm^2^ (Group 1) and 9 mW/cm^2^ (Group 2) in mild to moderate and advanced keratoconus at 12 months

	Mild to Moderate Keratoconus			Advanced Keratoconus		
	Group 1 (*n* = 23) (Mean ± SD)	Group 2 (*n* = 17) (Mean ± SD)	*P*	Group 1 (*n* = 14) (Mean ± SD)	Group 2 (*n* = 12) (Mean ± SD)	*P*
UCVA (LogMAR)	−0.12 ± 0.18	− 0.07 ± 0.17	0.354	−0.14 ± 0.26	−0.11 ± 0.25	0.803
BCVA (LogMAR)	−0.07 ± 0.11	−0.10 ± 0.13	0.515	−0.13 ± 0.14	−0.19 ± 0.16	0.37
K1 (D)	−0.01 ± 0.54	−0.22 ± 2.43	0.688	0.20 ± 0.95	− 1.08 ± 1.16	0.005
K2 (D)	0.05 ± 0.61	− 0.82 ± 1.13	0.003	0.14 ± 1.03	− 1.06 ± 1.37	0.019
AvgK (D)	0.01 ± 0.53	− 0.82 ± 1.10	0.003	0.16 ± 0.90	− 1.06 ± 1.21	0.007
Cycl (D)	0.06 ± 0.41	− 0.01 ± 0.60	0.619	− 0.06 ± 0.77	0.02 ± 0.67	0.757
AKf (D)	− 0.69 ± 1.36	−1.54 ± 1.47	0.067	− 0.94 ± 1.95	−2.27 ± 1.68	0.078
AKb (D)	3.89 ± 5.69	4.97 ± 4.80	0.531	3.40 ± 6.82	4.91 ± 3.82	0.504
Slf (D)	−0.28 ± 0.66	−0.64 ± 0.90	0.153	− 0.44 ± 1.27	−1.21 ± 0.56	0.065
Slb (D)	0.13 ± 0.25	0.12 ± 0.22	0.827	− 0.02 ± 0.25	−0.02 ± 0.36	0.94
Thin (μm)	−21.08 ± 23.60	−42.00 ± 28.61	0.016	− 18.21 ± 15.65	−38.58 ± 21.21	0.01
Vol (mm^3^)	−0.93 ± 1.46	−1.13 ± 2.83	0.777	− 1.18 ± 1.9	− 1.48 ± 2.73	0.747
CCT (μm)	− 11.82 ± 18.94	−21.05 ± 20.15	0.146	− 9.92 ± 18.77	−24.58 ± 23.86	0.092

*UCVA* = uncorrected visual acuity; *BCVA* = best-corrected visual acuity; *K1* = flat keratometry; *K2* = steep keratometry; *AvgK* = average keratometry; *Cyl* = topographic cylindrical value; *AKf* = apical keratoscopy front (Kmax); *AKb* = apical keratoscopy back; *SIf* = symmetry index front; *SIb* = symmetry index back; *Thin* = thinnest point of cornea; *Vol* = corneal volume; *CCT* = central corneal thickness; *D* = diopters

*P*: Paired t test; Values in bold are significant (*P* < 0.05)

Both groups showed no correlation between preoperative measurements (visual acuity, thinnest and central corneal thickness, corneal volume and AKf) and the change in AKf at 12 months (*P* > 0.05 for all) (Table 5). Regression analysis showed that the strongest relationship with the change in AKf was the type of treatment protocol (β = − 0.482, *P* = 0.005) (Table 6).

**Table 5:** Correlation between AKf change at 12-month and preoperative assessment variables

Ch_AKf		Preop UCVA	Preop BCVA	Preop Thin	Preop Vol	PreopCCT	Preop AKf
Group 1	R	0.004	0.041	0.188	−0.088	0.244	−0.320
	P	0.982	0.810	0.266	0.606	0.146	0.053
Group 2	R	0.031	0.059	−0.057	−0.136	−0.081	− 0.287
	P	0.872	0.762	0.768	0.481	0.678	0.131

*UCVA* = uncorrected visual acuity; *BCVA* = best-corrected visual acuity; *Thin* = thinnest point of cornea; *Vol* = corneal volume; *CCT* = central corneal thickness, *AKf* = apical keratometry front (Kmax); *Ch_AKf* = change in AKf at 12 months

**Table 6:** The results of regression analysis of model

	Beta	T	P	95% CI	
				Lower	Upper
(Constant)		1.290	0.202	−3.248	14.985
Gender	0.007	0.059	0.953	−0.766	0.813
Treatment Type*	− 0.482	−2.922	**0.005**	−2.699	− 0.503
Age	− 0.023	−0.183	0.855	−0.072	0.060
Preop AKf level (moderate or advanced)	−0.357	−1.976	0.053	−0.227	0.002
Pre BCVA	0.294	1.783	0.080	−0.269	4.637
Preop thinnest point	−0.022	−0.043	0.966	−0.045	0.043
Preop corneal volume	−0.225	−0.999	0.322	−0.294	0.098
Preop central corneal thickness	0.251	0.580	0.564	−0.026	0.047
Demarcation line	0.206	1.247	0.217	−0.004	0.016

a. Dependent Variable: Change in AKf at 12 months

*AKf* = apical keratoscopy front (Kmax); *BCVA* = best-corrected visual acuity; *CI* = confidence interval of difference

* Significant

## Discussion

Keratoconus progression, especially at younger ages, is aggressive and may not stop on its own. Improving the biomechanical strength of the cornea could be a greater benefit than waiting for patients to undergo corneal transplantation. CXL provides the effect of stopping the progression of keratoconus by increasing the degree of interfibrillar linkages through photopolymerization of riboflavin. The long-term results of patients undergoing standard CXL have proven the positive effect of this treatment on stabilizing the keratometric parameters [13–18]. However, this protocol is time-consuming and with their time- and cost-saving benefits, accelerated CXL protocols have been replaced in recent years in ophthalmology practice [3–7, 19].

The first article comparing the standard and accelerated techniques was published by Tomita et al. [7]. They reported no significant differences in postoperative changes in UCVA and BCVA, in the manifest refraction spherical equivalent or in the postoperative changes in the keratometric readings and the corneal biomechanical responses between the two procedures. According to their findings, a similar demarcation line was formed by three minutes 30 mW/cm^2^ accelerated CXL and conventional CXL. This first result encouraged ophthalmologists in the use of accelerated technique as a valid alternative for the standard protocol.

Since 2014, many published clinical trials, with at least one year of follow-up, were conducted to investigate the therapeutic effect of different types of accelerated CXL techniques (9 mW/cm^2^, 18 mW/cm^2^, 30 mW/cm^2^ and 45 mW/cm^2^), comparing them with the Dresden protocol [6–8, 20–23]. Even though most of these studies demonstrated successful clinical results, they could not achieve a different perspective. One of the reports that compared the two different types of protocols for accelerated CXL (30 mW/cm^2^ for four minutes and 18 mW/cm^2^ for five minutes) with a one-year follow-up in a larger cohort was recently published and showed no significant changes in spherical equivalent, visual and topographic results [9]. According to their findings, the authors concluded that both modalities of accelerated CXL (total doses of 7.2 J/cm^2^ and 5.4 J/cm^2^) exhibited comparable efficacy and applying higher energy for longer periods of time could reach satisfactory results in the stabilization of keratoconus progression following a mean of 12 months. In contrast to these findings, Choi et al. [22] reported that increasing the UV intensity (30 mW/cm2) and decreasing the irradiation time (to three minutes and 40 s) exhibited less topographical flattening than that of the conventional Dresden protocol. Another study published by Peyman et al. [24] supported Choi’s conclusion by investigating the stromal demarcation line depth in pulsed and continuous four minutes of accelerated CXL protocols. Peyman and associates reported that total fluence of 7.2 J/cm^2^ could not induce a deeper demarcation line in contrast to previous studies.

Although the results of all these studies are chaotic diversity, most of them indicate that the lower threshold of human corneas for irradiance is the main reason for Bunsen-Roscoe reciprocity law becoming invalid. Our observation corroborates with previous literature reports. Our study showed a strong positive relationship between CXL duration and topographic flattening. Although improvement in visual acuity was similar in both groups, the change in keratometric values K1, K2, AvgK, and AKf were significantly higher in 9 mW/cm^2^ for the 10 min group (P < 0.05 for all). Also, the change in SIf was significantly higher in the 9 mW/cm^2^ protocol.

Shetty et al. [10] compared the three accelerated CXL protocols (9 mW/cm^2^ for 10 min, 18 mW/cm^2^ for five minutes, and 30 mW/cm^2^ for three minutes) with the conventional CXL. In their prospective randomized interventional study, they reported that conventional CXL and 10 min accelerated CXL provided similar topographic improvement, while the effect of three minutes accelerated CXL was lower compared with the other groups at the end of 12 months. Furthermore, they highlighted the relationship between the flattening effect and energy of radiation, concluding that the efficiency decreases as the amount of energy increases. Although the authors provided a valuable contribution to the treatment capacity of accelerated CXL types, a few points in the study have overshadowed the results. Following CXL, improvements in topographical and aberrometry parameters indicate CXL’s functional success, even if its long term biomechanical impact in stabilizing progressive corneal ectasia is limited. Epstein et al. [25] reported that the Kmax value was the most important criterion in the follow-up of the progression of keratoconus. Many long-term studies that support this conclusion have stated that Kmax is the most commonly used parameter to determine keratoconus progression [16, 26]. The use of Kmax as an indicator of CXL success is the most important difference that distinguishes our work from Shetty and colleagues. We also verified Shetty’s preliminary results by changing methods and using Sirius topography. Since Sirius combines Placido disk topography with Sheimpflug tomography of the anterior segment, we could measure the large number of topographic parameters more precisely in a short period of time. Previous studies have found that these two devices differed significantly; Sirius showed good to excellent repeatability for all measured parameters, especially in healthy corneas [27–29]. In another study, Shetty and colleagues analyzed the effect of post collagen CXL haze on the measurement and repeatability of pachymetry and mean keratometry of four corneal topographers [30]. They reported that postoperative mean keratometry values measured with the Pentacam were affected by post-CXL haze. As a result of all these studies, we believe that the Sirius device gives more reliable results than the Pentacam for the tests performed after crosslinking.

In another retrospective case series supporting our findings, Toker and colleagues examined a series of 134 crosslinked eyes. They reported that although the standard and accelerated CXL (10 min, 9 mW/cm^2^; 4 min, 30 mW/cm^2^) results were similar in terms of keratometric stabilization, the four-minute method showed less topographic improvement. In this study, the demarcation line depth was similar between the standard and 10-min groups (266 mμ, and 273 mμ, respectively), and it was found 173 mμ in the four-minute group [11]. In our study, despite no correlation emerging between demarcation line depth and AKf, the change in corneal coma value was significantly higher in Group 2 (P < 0.05) where the demarcation line was deeper and closer to conventional epithelium-off CXL. Another consideration was that the concept of “efficacy” after CXL should be based mainly on its biomechanical impact because it is not a refractive procedure. The shallower demarcation line in the 18 mW/cm^2^ group may be due to the limited CXL activity because of insufficient oxygen in the environment due to increased oxygen consumption with high UVA intensity. Therefore, Mazzotta et al. [31] showed that the demarcation line reached an average of 280 mμ when they used 15 mW/cm^2^ UV-A power with pulsed light applied for 6 min.

In keratoconus, increased corneal HOAs further worsen optical quality and visual acuity [32]. By interpreting the changes in HOAs after CXL, it is possible to establish the benefit of CXL on optical and refractive functions. Studies have shown that in progressive keratoconus, there is an apparent reduction in HOAs after CXL [33]. However, to the best of our knowledge, there is no study in the literature comparing the corneal HOAs changes after accelerated CXL.

Alió and Shabayek [34] suggested using anterior corneal aberrations to evaluate keratoconus and reported that in eyes with keratoconus, coma-like aberrations were found to be significantly higher compared with normal eyes. In our study, we evaluated anterior corneal HOAs and compared their changes in the 6 mm zone. All anterior corneal aberrations demonstrated a significant reduction in both groups, in accordance with previous studies presenting improvement in HOAs [33, 35]. In their study with 96 eyes and a 12-month follow-up time, Greenstein et al. [33] reported a significant reduction in total anterior corneal HOAs, total coma, three order coma, and vertical coma. Caporossi et al. [35] also found a significant reduction in the total cornea HOAs and coma aberration from immediately after treatment up to 24 months in 44 eyes. In our study, further improvement was observed in coma values in the 9 mW/cm^2^ group (Group 2) at 12 months.

Another point to consider is the real association between the success rate of accelerated CXL types and preoperative keratoconus severity. Comparisons between the two subgroups revealed that there was no significant difference in terms of the mean change in UCVA and BCVA between the mild to moderate and advanced keratoconus at any examination. However, both subgroups showed a significant decrease in the mean changes in K2 and AvgK for the 9 mW/cm^2^ protocol, with K1 in advanced cases. Even though the differences did not reach a significant level, the 9 mW/cm^2^ for 10 min application had a greater flattening effect in the maximum keratometry in both subgroups.

Some limitations of this study are that there was no comparison with conventional CXL, this study had a short follow-up period, and the sample size was relatively small.

## Conclusions

Based on a mean follow-up time of 12 months, accelerated CXL using 10 min of UVA irradiance at 9 mW/cm^2^ showed better topographic improvements than five minutes of UVA irradiance at 18 mW/cm^2^, independent of keratoconus severity. Significant decreases were observed in HOAs in both groups, but in the 9 mW/cm^2^ group with the deeper demarcation line depth, coma aberrations improved further. Although shortening the duration of the procedure is a practical approach in terms of patient and physician comfort, optimum treatment protocol should be preferred to slow the progression.
